# Supplementary material for: Chromothripsis during telomere crisis is independent of NHEJ, and consistent with a replicative origin
Source: Genome Res. 2019 May;29(5):737–49. doi: 10.1101/gr.240705.118 (PMC6499312; doi:10.1101/gr.240705.118)
Supplement: Supplemental Material [file supp_gr.240705.118_Supplemental_file_1.zip › contigs/annotated_contigs/DB116/contig.2.DB116_length_694_mean_cov_10.4827089337.docx]

**DB116_length_694_mean_cov_10.4827089337**

```
CTTCCACTCCACTGGCAGGGCCATCTGCCAGGTCCACCCAGCCATAACCCAACCCAAGCCCATGCATGCTGACCAAGCCGTCCTTGTCT
 >chr8:37698417-37698748 + E=2e-187
CCGTACTCACCATATCCTGTCTCCCCAACCACCCCGGCCCCCAGCCCCACCCCAGCCATGCCCCCTGTCCTCATCACTGCTTCTGTGTC

TCCTACAGCTGCTGGCTGGTGTGGCGTCCAAGCCTTGGCGCCTTCTACATCCCTGTGGCTTTGATTCTGCTCATCACCTGGATCTATTT

CCTGTGCGCCGGGCTACGCTTACGGGGTCCTCTGGCACAGAACCCCAAGGCGGGCAACAGC|AGG|AGTGAGCACTCATTTGGAGAACT
                                                              >chr8:37720992-37721358 -
GAGTCCTCCCATGATGGCACTGCTTAAAATCCAAACCCAGAGTCAAGTCCAGAGGTCCTCGACCTGTGAGGCAAGTATGGTTTTTACAT
 E=2e-208
TTTTAAAAGTTCATACAACAAAATAAAACAAAACAAACAGACCAAAAAACCAAGGAAGAACATGTTAACAAAGGCTGTCTGTGGCCCGC

AAAGGCTCAAATATGTGCTATCTGGCCCTTTCCAGAAGACTTCATCAGTCAACAACCTGTTTTCACTGTAAATTGAAAACGGATTCCCT

GGCTGGGCACGGTGGCTCATGCCTGTAATCCCAGCACTTTGGGAGGCTGAGGCAGGCGGATCACCTGGGGTCA
```
